# The Effect of Oral Health Knowledge, Attitude, and Practice on Periodontal Status among Dental Students

**DOI:** 10.1055/s-0039-1697109

**Published:** 2019-10-18

**Authors:** Fatemah A. Ahmad, Mazen K. Alotaibi, Mohammad Abdul Baseer, Sanaa M. Shafshak

**Affiliations:** 1 Department of Preventive and Community Dentistry, Riyadh Elm University, Saudi Arabia; 2 Department of Periodontics, Prince Sultan Military Medical City, Riyadh, Saudi Arabia

**Keywords:** oral health knowledge, oral health attitudes and practice, periodontal status, dental students

## Abstract

**Objectives** The primary aim of the current study is to relate oral health knowledge, attitude, and oral hygiene practice with the periodontal condition of both undergraduate and postgraduate dental students.

**Materials and Methods** Data were collected through a combination of self-reported questionnaire and clinical examination. The estimated sample size was 246. Probing depths and clinical attachment loss were measured in interproximal sites, whereas the gingival index was calculated based on Ramfjord teeth. The Centers for Disease Control and the American Academy of Periodontology classification was used for periodontal diagnosis. The subjects were divided into three groups. Group 1 was composed of undergraduate, preclinical dental students, group 2 consisted of undergraduate clinical-year dental students, whereas group 3 included postgraduate residents.

**Results** A total of 296 dental students participated in this study. Significant differences were found among the groups in their oral health knowledge, attitude, and practice scores. Gingival disease was detected among most of the participants (99.2–100%) with significant differences between different educational levels (group 1 = 1.13, group 2 = 1.16, group 3 = 0.96, *p-* value = 0.001). Sixty percent of dental students were diagnosed with periodontal disease regardless of its severity. A positive correlation was established between oral health knowledge and attitude and oral hygiene practice. In addition, gingival inflammation severity and the severity of periodontal disease showed a positive correlation.

**Conclusions** This study highlighted the need to improve the oral health knowledge, attitude, and practice of dental students. Gingival and periodontal inflammation was highly prevalent among participants.

## Introduction

Oral health is defined as the ability to speak, taste, smell, smile, touch, swallow, and express emotions with confidence and without pain, disease, or discomfort of the craniofacial complex. 1 Oral health is considered an important component of general health that has been shown to influence the quality of life. Oral health may affect the individual’s appearance, social functions, and physical and psychological daily activities. 2 Periodontal health is a major component of oral health that concentrates on the prevention of inflammatory diseases in supportive tissue surrounding the teeth. 3

Oral hygiene practice can be defined as any effort performed by the individual to remove supragingival plaque. 4 Studies have shown that poor oral hygiene will lead to gingival inflammation and have established a linear relationship between plaque development and the presence of gingivitis. 5 The development of gingivitis had been linked to the development of periodontitis. 6 Therefore, cleaning the oral cavity is essential because it removes bacterial accumulation and prevents periodontal disease progression. 5

Dental students have an important role in oral health care promotion during their educational years and after graduation. 7 Teaching is considered effective if it leads to profound changes in students’ knowledge, attitude, and practice regarding their personal oral health. Dental students’ oral health knowledge, attitude, and practice are important because it affects their capacity to translate information to their patients. 8

Several studies have evaluated the oral health knowledge, attitude, and oral hygiene practice among health care providers 9 10 11 12 ; however, most of these studies concentrated on oral health attitudes and practice, and part of them evaluate the data together without separating oral health knowledge, attitude, and oral hygiene practices into separate domains.

When comparing the oral health knowledge among dental and medical students, 96.6% of dental students and 88.6% of medical students knew the purpose of tooth brushing. Dental students were more aware of the importance of flossing than medical students were (88 vs. 64%). On the other hand, almost all dental and medical students agreed that a soft toothbrush was preferable to a hard one. 13 Male dental students were found to have adequate knowledge in some aspects, such as the role of fluoride in prevention of dental caries, and limited knowledge in others, for example, the risk of periodontal disease, in a study that was performed in Kuwait. 9

Investigating their attitude toward oral health and oral hygiene practices illustrated that dental students scored higher than medical students. 14 Eighty-nine percent of dental students, compared with 54% medical students, believed that regular dental visits were important. However, 81% of dental and 83% of medical students had never used dental floss. 8 A study conducted in Spain comparing third-year medical and dental students found more positive oral health perception among dental students than among their peers in medical school. The majority of dental students visited the dentist every 6 months (29.5%) or annually (38.6%), whereas medical students’ visits (55.8%) depended on the level of pathology. 12

Other studies concentrated on the changes that occurred during the dental educational progress. When assessing the oral health knowledge, attitude, and practice of dental students, it was noted that the mean scores increased significantly as the dental students progressed in their educational program. 15

Although most of these are questionnaire-based studies, a few involved the dental examination in their investigation. 13 14 16 However, according to the best of our knowledge, no study related oral health knowledge, attitude, and practice with the periodontal condition. Therefore, the primary aim of this study was to relate oral health knowledge, attitude, and oral hygiene practice with periodontal condition among both undergraduate and postgraduate dental students.

## Materials and Methods

This is a cross-sectional study conducted at Riyadh Elm University, Kingdom of Saudi Arabia, during a period ranging from October 2017 to May 2018. Ethical approval was granted by the ethical committee of the research center in Riyadh Elm University (ethical approval number: RC/IRB/2016/530). Power analysis was conducted by G* power calculator, and the sample size was estimated to be 246.

All dental students in Riyadh Elm University had been invited to participate in this study by either e-mail or direct communication or indirectly through their supervisors. No gender predisposition was applied in this study. The exclusion criteria included third molars, any medical condition that affected the subject’s dexterity, uncontrolled diseases that affected the periodontal condition, subjects with orthodontic appliances or complex prosthodontic prosthesis or implants, and those who were pregnant.

Accordingly, appointments were arranged with every participant who agreed to be involved in this study and met the above-mentioned criteria. Consent forms were signed and confidentiality was maintained. During this appointment, the participant was asked to fill out a questionnaire that was used to assess oral health knowledge, attitude, and oral hygiene practice. The questionnaire was constructed based on previous articles and American Dental Association recommendations after the validation. 7 17 18 19 The clinical examination was performed by taking the measurements of probing depth (PD) and clinical attachment loss (CAL) on interproximal sites (mesiobuccal and distobuccal), including all maxillary and mandibular teeth, with the exception of the third molars, to determine the periodontal status by using a University of North Carolina (UNC) periodontal probe and intraoral mirror. The criteria developed by the Centers for Disease Control and the American Academy of Periodontology (CDC-AAP) were followed to identify subjects with periodontitis ( Table 1 ). 16 20 21 The gingival index (GI) was calculated based on the Ramfjord teeth index. 22 The patients were classified as having healthy gingiva if their GI score was <0.1. Mild gingivitis was diagnosed if GI = 0.1—1, whereas moderate gingivitis was identified with GI = 1.1—2. Individuals with GI = 2.1—3 were categorized as subjects with severe gingivitis.

**Table 1:** CDC-AAP criteria of periodontal disease

Periodontal diagnosis	Criteria
Mild	≥3 mm CAL in two or more interproximal sites and two or more sites with ≥ 4 mm PD
	Or one site with PD ≥ 5 mm﻿
Moderate	Two or more interproximal sites with ≥ 4 mm CAL (not on the same tooth)
	Or two or more sites with ≥ 5 mm PD (not on the same tooth)
Severe	Two or more interproximal sites with ≥ 6 mm CAL (not detected on the same tooth) and one or more interproximal sites with ≥ 5 mm PD
Abbreviations: CAL, clinical attachment loss; CDC-AAP, Centers for Disease Control and the American Academy of Periodontology; PD, probing depth.	

The study participants were divided into three groups. In this university, in the first 3 years, the students attend basic science lectures and laboratory courses. Their oral health knowledge depends mainly on their background; these preclinical-year students were considered a control group (group 1). During the fourth to sixth years, dental students are involved in different dental specialty courses and start to treat patients in the clinics. These clinical-year dental students were included in group 2. Since Riyadh Elm University adopted a postgraduate program, postgraduate residents were categorized as group 3.

### Intra-Examiner Calibration

At the beginning of the study, calibration of the examiner (FA) was done on seven patients. Examinations of 492 sites were performed and were repeated after 2 weeks. A Hu-Friedy UNC probe was used to take the measurements. The reliability test results illustrated that absolute agreement was 71.3% and agreement within ± 1 mm was 91.8% for the PD (Cohen’s kappa = 0.69). When measuring CAL, absolute agreement was 91.4%, agreement within ± 1 mm was 96.3%, and Cohen’s kappa equaled 0.68.

### Statistical Analysis

Data was entered and analyzed using SPSS version 20. The questions were recoded, and the correct answers had a code of 1, whereas all incorrect answers had a code of 0. Based on that, a scale was developed. The knowledge domain score had a range of zero to seven, whereas the attitude and practice domains had a range of zero to six. Differences in groups were assessed using the chi-squared test. Correlation was evaluated by using Spearman’s test. Statistical significance was considered when *p* -value < 0.05.

## Results

A total of 302 individuals participated in the study. Of these, six participants were excluded due to either incomplete data that prevented statistical analysis performance or uncontrolled diseases that increased the risk of periodontal disease presence. This yielded a final sample size of 296 students, of whom 55.1% were males and 44.9% were females. Among the participants, 30.1% were in group 1, 44.9% were in group 2, and 25% were in group 3. Descriptive data are shown in Table 2 .

**Table 2:** Characteristics of the study participants (n = 296)

Characteristics			*n*	%
Gender	Male		163	55.1%
	Female		133	44.9%
	Total		296	100.0%
Academic education	Undergraduate	Group 1	89	30.1%
		Group 2	133	44.9%
		Total	222	75.0%
	Group 3		74	25.0%
	Total		296	100.0%
Smoking status	Regular smoker		45	15.2%
	Former smoker		12	4.1%
	Never smoker		218	73.6%
	Occasional smoker		21	7.1%
	Total		296	100.0%

A comparison was made between the three groups in oral health knowledge, attitude, and oral hygiene practice. As the students progressed in their education, their scores increased significantly ( *p-* value = 0.000, 0.017, 0.000, respectively) ( Table 3 ).

**Table 3:** Comparison of knowledge, attitude, and practice ranks between preclinical- and clinical-level undergraduate and postgraduate students

Variables		*n*	Mean	SD	Mean rank	Chi-square	*p-* Value
Knowledge	Group 1	89	2.97	1.27	99.66	43.628	0.000
	Group 2	130	3.98	1.09	160.98		
	Group 3	73	4.21	1.22	177.82		
Attitude	Group 1	89	4.22	1.18	132.47	8.146	0.017
	Group 2	133	4.44	1.03	147.73		
	Group 3	74	4.72	1.09	169.16		
Practice	Group 1	89	2.54	1.17	102.25	39.506	0.000
	Group 2	133	3.60	1.17	170.74		
	Group 3	74	3.53	1.28	164.15		
Abbreviation: SD, standard deviation.							

Clinical examination indicated that group 2 had the highest mean of gingival inflammation severity (mean = 1.16). On the contrary, group 3 showed the healthiest gingiva (mean = 0.96). The difference between the three groups was statistically significant ( *p* -value = 0.001) ( Table 4 ).

**Table 4:** Comparison of gingival index score among preclinical, clinical, and postgraduate students

Gingival index	*n*	Mean	SD	Mean ranks	Chi-square	df	*p-* Value
Group 1	89	1.13	0.38	152.11	13.89	2	0.001
Group 2	133	1.16	0.40	163.34			
Group 3	74	0.96	0.41	117.49			
Total	296	1.10	0.40				
Abbreviation: SD, standard deviation.							

Among the participating dental students, group 2 presented with more mild, moderate, and severe periodontitis than group 1 and group 3 (42.4, 51.9, and 50%, respectively) ( Table 5 ).

**Table 5:** Periodontal diagnosis among participating dental students based on educational level

students				Periodontal diagnosis			Total
			Mild	Moderate	Severe	No periodontitis	
	Group 1	*n*	29	23	1	36	89
		(%)	31.5%	29.1%	16.7%	30.3%	30.1%
	Group 2	*n*	39	41	3	50	133
		(%)	42.4%	51.9%	50.0%	42.0%	44.9%
	Group 3	*n*	24	15	2	33	74
		(%)	26.1%	19.0%	33.3%	27.7%	25.0%
	Total	*n*	92	79	6	119	296
		(%)	100%	100.0%	100.0%	100.0%	100.0%

When correlating all the studied variables, a significant positive correlation was found between the severity of gingivitis and the periodontal diagnosis. A significant positive correlation was also detected between oral health knowledge, attitude, and oral hygiene practices. On the other hand, the correlation between oral health knowledge, attitude, and oral hygiene practice with periodontal status did not reach a significant level ( *p* = 0.79, 0.61, 0.61, respectively) ( Table 6 ).

**Table 6:** Spearman’s correlation among gingivitis severity, periodontal diagnosis, and oral health knowledge, attitude, and oral hygiene practice

		Gingivitis severity	Periodontal diagnosis	Knowledge	Attitude	Practice
Gingivitis severity	Correlation coefficient	1.000	0.381 ^a^	–0.059	0.043	–0.065
	Sig. (2-tailed)	—	0.000	0.317	0.461	0.264
	*n*	296	296	292	296	296
Periodontal diagnosis	Correlation coefficient	0.381 ^a^	1.000	–0.016	0.030	–0.030
	Sig. (2-tailed)	0.000	—	0.790	0.613	0.611
	*n*	296	296	292	296	296
Knowledge	Correlation coefficient	–0.059	–0.016	1.000	0.154 ^a^	0.406 ^a^
	Sig. (2-tailed)	0.317	0.790	—	0.008	0.000
	*n*	292	292	292	292	292
Attitude	Correlation coefficient	0.043	0.030	0.154 ^a^	1.000	0.151 ^a^
	Sig. (2-tailed)	0.461	0.613	0.008	.	0.009
	*n*	296	296	292	296	296
Practice	Correlation coefficient	–0.065	–0.030	0.406 ^a^	0.151 ^a^	1.000
	Sig. (2-tailed)	0.264	0.611	0.000	0.009	—
	*n*	296	296	292	296	296
**^a^** Correlation is significant at the 0.01 level (2-tailed).						

## Discussion

Periodontal disease is one of the most common oral diseases that causes global burden worldwide. It is highly prevalent and considered a major public health problem in several countries. 23 Although dental plaque is considered a primary etiologic factor in the development of periodontal diseases, 24 alteration of the inflammatory response course may accelerate periodontal tissue destruction. This can occur with uncontrolled diabetes, 25 smoking, 26 psychosocial stress, 27 and fluctuation in hormonal levels. 28

Dental students are considered future oral health providers. To motivate their patients to implement good oral health, they should be adequately self-motivated.

There was a statistically significant improvement among preclinical, clinical-year undergraduate students, and postgraduate residents in oral health knowledge and attitude scores. Similarly, studies conducted in Kuwait, Turkey, Kerala, and Croatia illustrated that as students progressed through their dental educational program, their oral health knowledge, attitude, and oral hygiene practice improved. 10 11 15 29 However, postgraduate residents had not been involved in previous studies. Interestingly, the oral hygiene practice score of postgraduate residents was statistically lower than among the undergraduate students. This may be due to a higher stress level, which might be a modifying factor in periodontal diseases.

The prevalence of gingival disease was found to be high among the studied population, ranging between 99.2 and 100%. Unfortunately, only limited studies reported the gingival condition among dental students. The assessment methodology was highly variable among them to compare the results. Bleeding on probing (BOP) was one of the highly used indicators to assess the presence of gingival diseases. Based on that parameter, in accordance with the presented findings, a study conducted among medical and dental university students showed that only 26% of subjects presented with healthy gingiva (BOP < 20% of the sites), with no significant difference between males and females. 30 In addition, Lucena et al found that a low percentage (0.8%) of university students, mainly those who attend dentistry, pharmacy, and nursing courses, presented with healthy gingiva. 31 In the United Arab Emirates, 21.5% of dental students presented with BOP ≤25%. 32 Comparing the gingival condition between medical and nonmedical categories, nonmedical students presented with significantly more BOP than medical field students did. 33 In the current study, it was noticed that gingival health improved significantly as the students progressed in their educational program form undergraduate to postgraduate education. This may be due to their acquisition of knowledge that led to behavioral changes.

Periodontal disease was detected in 60% of the dental students regardless of severity. In this investigation, 31% of the dental students suffered from mild periodontitis, whereas 27% were diagnosed with moderate periodontitis. Marulanda et al illustrated that 8% of participants were diagnosed with moderate periodontitis, less than in the current study. Although Marulanda et al did not have subjects with severe periodontitis, 30 the current data indicated that 2% of participants were diagnosed with severe periodontitis. These results were even higher than the prevalence detected in the general population in Turkey (11—40%). 34 In accordance with the current findings, a high prevalence of periodontal alternation (99.2%) was found in Brazil. 31 Although periodontal disease is considered a multifactorial disease, this high prevalence among dental students needs to be investigated further. All contributing factors should be studied to control the prevalence and severity of periodontal diseases.

Dental students have been proven to suffer from high levels of stress compared with other specialties. This stress level increases as they progress in the educational program, especially in their final years. 35 36 In addition, subjects with type D personality—people who tend to be alone and depressed and express negative emotions—were more likely to present with periodontal disease. 37 Although significant improvement was detected in oral health knowledge, attitude, and oral hygiene practice as the students progressed in their education in this study, this was not reflected in the periodontal diagnoses.

In an attempt to investigate the relationship between different variables, we found that as oral health knowledge increased, students developed a positive attitude and performed better oral hygiene. This correlation was statistically significant. In addition, a statistically significant positive correlation between gingival inflammation severity and periodontal disease severity was established. This agrees with previously proven evidence that gingivitis is a risk factor for developing periodontitis. 38

## Strengths and Limitations

The CDC-AAP classification was based on both PD and CAL. Although CAL may be considered more accurate than PD, depending on CAL alone could include healthy reduced periodontium or CAL due to other causes than periodontal disease in the periodontitis category. In a similar pattern, depending on PD alone results in underestimation of periodontitis prevalence, especially in older persons. Therefore, a combination of both CAL and PD will result in more accurate diagnoses. 20 39

The periodontal examination was performed by a single examiner, which increased the reliability of the measurements taken and reduced the errors and variations. On the other hand, the CDC-AAP classification was based mainly on interproximal site measurements, due to the assumption that these sites were the most affected. This may lead to underestimation of periodontal disease. Neither BOP nor furcation involvement was recorded, even though such measurements may provide additional information that could help diagnose periodontal disease accurately, based on other case definitions. 40

Because this study is a cross-sectional study, the observed changes cannot be directly connected to the curriculum, but it can be used as a good indicator of needed changes in both undergraduate and postgraduate educational programs. 11 In addition, this study was based on collecting samples from one university. Multicenter studies are needed to generalize the results. Including female dental students may be considered a weak point because hormonal fluctuation was not considered.

## Conclusions and Recommendations

In light of these results, although a significant improvement was detected in oral health knowledge, attitude, and oral hygiene practice as the dental students progressed from undergraduate preclinical years to postgraduate specialty programs, the scores were considerably lower than what was expected.

High prevalence of gingival inflammation was detected among dental students. In addition, more than half of the participants presented with periodontal disease, regardless of severity.

It can be stated that improving oral health knowledge leads to better attitude and preferable oral hygiene practice. Furthermore, a positive correlation was established between gingivitis severity and periodontal inflammation severity.

As health care providers consider a role model for their families, friends, and patients, teaching them the necessary skills to attain oral health is imperative. Educators and policy makers need to assess the educational programs further to bridge the gaps for better oral health care. Therefore, it is suggested that oral health and preventive dentistry courses should be stressed in the both undergraduate curriculum and in specialty postgraduate programs. In addition, such topics should start early in dental training.

The periodontal disease burden can be reduced with intensive oral health promotion campaigns. In addition, establishing an oral health clinic for annual examination and providing needed treatment for dental students are considered a valuable step in improving oral health. One step to emphasize the role of such a clinic is to consider the annual examination as one of the requirements needed to progress in a dental training program.

## References

[1] FDI World Denal Federation FDI's definition of oral health www.fdiworlddental.org/oral-health/fdi-definition-of-oral-health Updated 2016

[2] Gomes A S Abegg C Fachel J M Relationship between oral clinical conditions and daily performances Braz Oral Res 2009 23 01 76 81 19488476 10.1590/s1806-83242009000100013

[3] Lang N P Bartold P M Periodontal health J Clin Periodontol 2018 45 20 S9 S16 29926485 10.1111/jcpe.12936

[4] Lindhe J Lang N Clinical Periodontology and Implant Dentistry 6thed.UK Wiley Blackwell 2017

[5] Loe H Theilade E Jensen S B Experimental gingivitis in man J Periodontol 1965 36 177 187 14296927 10.1902/jop.1965.36.3.177

[6] Page R C Schroeder H E Pathogenesis of inflammatory periodontal disease. A summary of current work Lab Invest 1976 34 03 235 249 765622

[7] Hosing A Hiremath A M Vadavadagi V Bansal A Kahar A Oral hygiene practices in dental students JOHCD 2016 10 01 30 34

[8] Kumar S Motwani K Dak N Balasubramanyam G Duraiswamy P Kulkarni S Dental health behaviour in relation to caries status among medical and dental undergraduate students of Udaipur district, India Int J Dent Hyg 2010 8 02 86 94 20522130 10.1111/j.1601-5037.2008.00346.x

[9] Al-Ansari J Honkala E Honkala S Oral health knowledge and behavior among male health sciences college students in Kuwait BMC Oral Health 2003 3 01 2 12735791 10.1186/1472-6831-3-2 PMC156614

[10] Ali D A Assessment of oral health attitudes and behavior among students of Kuwait University Health Sciences Center J Int Soc Prev Community Dent 2016 6 05 436 446 27891310 10.4103/2231-0762.192943 PMC5109858

[11] Badovinac A Božić D Vučinac I Vešligaj J Vražić D Plancak D Oral health attitudes and behavior of dental students at the University of Zagreb, Croatia J Dent Educ 2013 77 09 1171 1178 24002855

[12] Cortes F J Nevot C Ramon J M Cuenca E The evolution of dental health in dental students at the University of Barcelona J Dent Educ 2002 66 10 1203 1208 12449215

[13] Kumar H Behura S S Ramachandra S Nishat R Dash K C Mohiddin G Oral health knowledge, attitude, and practices among dental and medical students in Eastern India - a comparative study J Int Soc Prev Community Dent 2017 7 01 58 63 28316951 10.4103/jispcd.JISPCD_30_17 PMC5343685

[14] Jella R K Pratap K Padma T M Kalyan V S Vineela P Varma L S Oral health attitude and behavior among health-care students in a teaching hospital, Telangana State: a cross-sectional Study Indian J Dent Sci 2016 8 242 245

[15] Ahamed S Moyin S Punathil S Patil N A Kale V T Pawar G Evaluation of the oral health knowledge, attitude and behavior of the preclinical and clinical dental students J Int Oral Health 2015 7 06 65 70 PMC4479777 26124603

[16] Petruţiu S A Stratul S I Soancă A The impact of some behavioral aspects on periodontal disease in a group of Romanian students - an epidemiological survey Rev Epidemiol Sante Publique 2014 62 06 367 375 25454747 10.1016/j.respe.2014.08.006

[17] American Dental Association A. Floss/Interdental Cleaners https://www.ada.org/en/member-center/oral-health-topics/flossUpdated20 10 2017

[18] American Dental Association A. Toothbrushes https://www.ada.org/en/member-center/oral-health-topics/toothbrushesUpdated18 9 2017

[19] Komabayashi T Kwan S Y Hu D Y Kajiwara K Sasahara H Kawamura M A comparative study of oral health attitudes and behaviour using the Hiroshima University - Dental Behavioural Inventory (HU-DBI) between dental students in Britain and China J Oral Sci 2005 47 01 1 7 15881222 10.2334/josnusd.47.1

[20] Page R C Eke P I Case definitions for use in population-based surveillance of periodontitis J Periodontol 2007 78 07 1387 1399 10.1902/jop.2007.060264 17608611

[21] Eke P I Page R C Wei L Thornton-Evans G Genco R J Update of the case definitions for population-based surveillance of periodontitis J Periodontol 2012 83 12 1449 1454 22420873 10.1902/jop.2012.110664 PMC6005373

[22] Löe H The Gingival Index, the Plaque Index and the Retention Index Systems J Periodontol 1967 38 06Suppl610 616 10.1902/jop.1967.38.6.610 5237684

[23] Petersen P E Ogawa H The global burden of periodontal disease: towards integration with chronic disease prevention and control Periodontol 2000 2012 60 01 15 39 22909104 10.1111/j.1600-0757.2011.00425.x

[24] Haffajee A D Socransky S S Microbial etiological agents of destructive periodontal diseases Periodontol 2000 1994 5 78 111 9673164 10.1111/j.1600-0757.1994.tb00020.x

[25] Bernick S M Cohen D W Baker L Laster L Dental disease in children with diabetes mellitus J Periodontol 1975 46 04 241 245 1055217 10.1902/jop.1975.46.4.241

[26] Mirbod S M Ahing S I Pruthi V K Immunohistochemical study of vestibular gingival blood vessel density and internal circumference in smokers and non-smokers J Periodontol 2001 72 10 1318 1323 11699472 10.1902/jop.2001.72.10.1318

[27] Genco R J Ho A W Grossi S G Dunford R G Tedesco L A Relationship of stress, distress and inadequate coping behaviors to periodontal disease J Periodontol 1999 70 07 711 723 10440631 10.1902/jop.1999.70.7.711

[28] Mombelli A Gusberti F A van Oosten M A Lang N P Gingival health and gingivitis development during puberty. A 4-year longitudinal study J Clin Periodontol 1989 16 07 451 456 2768539 10.1111/j.1600-051x.1989.tb01674.x

[29] Peker K Uysal O Bermek G Dental training and changes in oral health attitudes and behaviors in Istanbul dental students J Dent Educ 2010 74 09 1017 1023 20837744

[30] Marulanda A M Coral D Sabogal D Serrano C Periodontal conditions of Colombian university students aged 16 to 35 Braz Oral Res 2014 28 28 24878670 10.1590/1807-3107bor-2014.vol28.0009

[31] Lucena R Menezes K Cavalcanti A Granville-Garcia A Uchôa Lins R Oral hygiene habits, periodontal status and need for treatment in university students Rev Odonto Ciênc 2012 27 04 289 293

[32] Rahman B Kawas S A The relationship between dental health behavior, oral hygiene and gingival status of dental students in the United Arab Emirates Eur J Dent 2013 7 01 22 27 23408498 PMC3571505

[33] Sharda A J Shetty S Relationship of periodontal status and dental caries status with oral health knowledge, attitude and behavior among professional students in India Int J Oral Sci 2009 1 04 196 206 20690423 10.4248/IJOS09061 PMC3733598

[34] Ilhan D Oktay I Nur B Percentage and severity of periodontal diseases in Turkish adults aged 35+ years, 2009-10 J Public Health Dent 2017 77 04 325 333 28369953 10.1111/jphd.12211

[35] Tangade P S Mathur A Gupta R Chaudhary S Assessment of stress level among dental school students: an Indian outlook Dent Res J (Isfahan) 2011 8 02 95 101 22013469 PMC3177400

[36] Ahmad F A Karimi A A Alboloushi N A Al-Omari Q D AlSairafi F J Qudeimat M A Stress level of dental and medical students: comparison of effects of a subject-based curriculum versus a case-based integrated curriculum J Dent Educ 2017 81 05 534 544 28461630 10.21815/JDE.016.026

[37] Mizutani S Ekuni D Yamane-Takeuchi M Type D personality and periodontal disease in university students: a prospective cohort study J Health Psychol 2018 23 05 754 762 27694404 10.1177/1359105316668668

[38] Lang N P Schätzle M A Löe H Gingivitis as a risk factor in periodontal disease J Clin Periodontol 2009 36 10 3 8 10.1111/j.1600-051X.2009.01415.x 19432625

[39] Beck J D Koch G G Offenbacher S Attachment loss trends over 3 years in community-dwelling older adults J Periodontol 1994 65 08 737 743 7965548 10.1902/jop.1994.65.8.737

[40] Eke P I Dye B A Wei L Update on prevalence of periodontitis in adults in the United States: NHANES 2009 to 2012 J Periodontol 2015 86 05 611 622 25688694 10.1902/jop.2015.140520 PMC4460825

